# Staged Resection with Temporizing VAC and Local Recurrence for Soft Tissue Sarcomas: A Multi-Institutional Review

**DOI:** 10.3390/cancers18121984

**Published:** 2026-06-18

**Authors:** Chloe Chose, Thomas Karadimas, Lucy Hederick, Anthony M. Griffin, Veena Jajoo, Caleb Cummings, Joseph Connolly, Thien Huong Huynh, Erik T. Newman, Kevin A. Raskin, Peter C. Ferguson, Jay S. Wunder, David Joyce, Odion Binitie, Rahul Mhaskar, Santiago A. Lozano-Calderon, Kim Tsoi, Alexander L. Lazarides

**Affiliations:** 1 Morsani College of Medicine, University of South Florida, Tampa, FL 33602, USA; 2 Division of Orthopaedic Oncology, Massachusetts General Hospital, Boston, MA 02114, USA; 3 Division of Orthopaedic Surgery, Mount Sinai Hospital, Toronto, ON M5G 1X5, Canada; 4 Department of Sarcoma, Moffitt Cancer Center, Tampa, FL 33612, USA

**Keywords:** soft tissue sarcoma, orthopaedic oncology, staged resection

## Abstract

**Background/Objectives**: Staged resection with temporizing vacuum-assisted closure (VAC) has been adopted to facilitate margin assessment and local control of soft tissue sarcomas (STS). Accordingly, the objective of this study is to compare rates of local recurrence (LR) among superficial STS patients treated with and without temporizing VAC therapy. **Methods**: We conducted a multi-institutional retrospective cohort study of superficial STS patients treated at three tertiary care centers. Patients were matched by grade, and descriptive statistics and multivariate binary logistic regression were performed. **Results**: After matching, 314 superficial STS patients were included in analysis. VAC patients were more likely to present with a history of prior unplanned excision (*p* = 0.024), with recurrent disease (*p* = 0.004), harbor a diagnosis of myxofibrosarcoma (*p* < 0.001), and have positive initial surgical margins (*p* < 0.001). After multivariate analysis, VAC use remained independently associated with increased LR rates (OR = 4.117; *p* = 0.003). **Conclusions**: In this multi-institutional analysis of superficial STS patients, staged resection with temporizing VAC was not associated with improved LR rates. Rather, the VAC cohort represented distinctly higher-risk tumor biology and complex oncologic presentations, in whom the risk of LR may not be fully mitigated by surgical staging alone.

## Simple Summary

This multi-institutional retrospective cohort study evaluated whether staged resection with temporizing vacuum-assisted closure (VAC) is associated with local recurrence. A total of 314 patients were grade-matched across three tertiary care centers. Patients who underwent staged resection more frequently exhibited higher oncologic risk factors, including history of unplanned excision, recurrent disease, myxofibrosarcoma histology, and positive initial surgical margins. On multivariate analysis, temporizing VAC use remained independently associated with increased local recurrence rates. Overall, staged resection with VAC did not confer improved local control, but rather characterized a cohort of more aggressive tumor biology and complex presentations for which staged resection may not fully mitigate.

## 1. Introduction

Soft tissue sarcomas (STS) are a heterogenous group of cancers marked by variability in biology, behavior, presenting features, and outcomes, which makes these tumors uniquely complex to manage [1]. Local recurrence (LR) remains a feared and clinically significant event in the disease course of STS, occurring at a median of 10.8 to 19 months after resection [2,3], with 80% recurring within the first 3 years [4]. LR is associated with higher rates of limb amputation and morbidity [5]. Studies also suggest an increased risk of distant metastasis and decreased overall survival with LR [6]. As such, local control is a central tenet in the treatment of these tumors, typically achieved by wide surgical resection with negative margins [7]. Positive surgical margins have been independently associated with worse overall survival and disease-specific survival [8]. Even after LR of a tumor, positive margins following re-resection yielded poorer re-recurrence and overall survival outcomes [9].

The gold standard for assessment of resection margins is postoperative pathologic assessment. However, this process can be time consuming, particularly in cases with complex anatomy or infiltrative growth patterns [10]. In response, staged resection with temporizing vacuum-assisted closure (VAC) has been adopted as a strategy to defer definitive soft tissue reconstruction until final margin status has been confirmed. Peri-operative VAC use in oncologic resection in general has been shown to be safe and harbor no increased risk of recurrence [11]. Its role in staged resection permits planned re-excision in cases of positive margins prior to definitive wound closure, with the intent of optimizing local control while minimizing reconstructive morbidity [12]. It has been described in management of malignant breast tumors [12] but has garnered special attention in the management of STS, particularly those complex reconstructive needs and high oncologic risk requiring careful margin assessment. Consistent with this approach, the National Comprehensive Cancer Network currently recommends the staging of infiltrative tumors, including myxofibrosarcoma and dermatofibrosarcoma protuberans (DFSP) [13]. Nevertheless, formal evidence-based guidelines defining the role of staged resection with VAC across the broader spectrum of STS remain lacking.

When compared with traditional single-stage excision for myxofibrosarcoma, VAC therapy was found to have lower LR rates (5.6% vs. 28.6%, *p* = 0.048) [14] and lower overall mortality (11.1% vs. 32%, *p* = 0.015) [15]. It also harbored an acceptable complication profile [16], similar cost profile [15], and similar patient-reported outcomes [17]. A separate study demonstrated that a staged approach, albeit without VAC therapy, obtained higher rates of negative final margins and resulted in significantly lower LR rates [18]. On the other hand, a separate institution found a 90% LR-free survival among superficial myxofibrosarcoma tumors treated with single-stage excision [19], revealing conflicting evidence as to the impact of VAC therapy on mitigating oncologic outcomes.

To date, the existing literature evaluating temporizing VAC therapy in STS largely focuses on individual histologic subtypes, notably myxofibrosarcoma, often within single-institution cohorts. Although these studies suggest potential benefits for local control in select tumors, their findings may not be generalizable across the heterogeneous population of superficial STS. Accordingly, the objective of this multi-institutional retrospective study is to compare rates of LR among patients with superficial STS treated with and without temporizing VAC therapy.

## 2. Materials and Methods

### 2.1. Patient Selection

We conducted a multi-institutional retrospective cohort study of patients treated surgically for STS at three tertiary referral centers between 2018 and 2024. Two of these institutions routinely employ staged resection with temporizing VAC when final margin status is uncertain, whereas the third institution does not and thus served as a comparison cohort. Patients in the VAC cohort were exclusively drawn from the two institutions that employ this strategy, while all patients from the third institution comprised the non-VAC comparison group.

Eligible patients included adults with pathologically confirmed STS who underwent surgical resection and had a minimum of one year of follow-up from the date of surgery. To avoid survivorship bias, patients who experienced LR or death within the first postoperative year were retained in the analysis. Deep-seated tumors involving the deep fascia were excluded to improve cohort homogeneity and limit confounding related to tumor depth, reconstructive complexity, and radiation exposure. Accordingly, the final analytic cohort was restricted to patients with superficial STS.

### 2.2. Data Collection

Demographics, tumor information, surgical and treatment-related variables, and outcomes were collected from electronic health record information. Pathology reports were reviewed for tumor grade and margin status. The primary outcome of interest was LR, defined as radiographic or clinical evidence of tumor recurrence at the operative site, confirmed either by imaging or by histopathologic analysis following re-excision. Due to limited sample size, race was recoded into two categories: White and non-White. Presenting status was categorized into primary tumor, locally recurrent tumor, and metastatic tumor to distant soft tissue site. Given low numbers of metastatic tumors (*n* = 3), locally recurrent and metastatic tumors were recoded as recurrent tumors for analysis.

### 2.3. Statistical Methods

Propensity score matching was attempted; however, adequate balance could not be achieved due to baseline differences between cohorts. Grade was then selected as the matching variable, because it is the most consistently identified independent predictor of LR across the broad heterogeneous STS histologic subtypes [20]. Matching on histologic subtype was not feasible due to the large number of subtypes with small cell sizes (Tables S1 and S2). After optimal manual matching of cases with randomized controls, the final analytic sample included an equal number of cases and controls.

Descriptive statistics were calculated for all variables. Continuous variables are presented as mean and standard deviation, while categorical variables are presented as frequencies and percentages. Because grade matching was used to construct comparable cohorts rather than establish analyzable matched pairs, observations were treated as independent in subsequent analyses. The primary variable of interest, temporizing VAC, and the dichotomous outcome variable, LR, were evaluated using Mann–Whitney U test for continuous variables using standard tie corrections. Pearson’s Chi-Square test or Fisher’s Exact test was used for categorical variables when expected cell counts were low. Univariate analyses were conducted to assess the association between each covariate and LR. Variables that were statistically significant in univariate analyses were subsequently included in a multivariate binary logistic regression model to identify independent associations of LR. Results are reported as odds ratios (OR) with 95% confidence intervals (CI). Finally, Kaplan–Meier analysis was performed to capture time-to-local recurrence, and survival curves were compared using log-rank tests. A *p*-value of <0.05 was considered statistically significant. All analyses were conducted using SPSS version 30.

## 3. Results

A total of 1242 patients with STS were identified across the three institutions. After applying inclusion criteria and excluding deep tumors, 519 patients with superficial STS were eligible for analysis. Manual 1:1 matching then resulted in a final matched cohort of 314 patients, with equal numbers among VAC and control groups.

### 3.1. Unmatched Cohort Analysis

The unmatched cohort analysis included 519 patients. Patients who received temporizing VAC were older on average (63.86 ± 15.62 vs. 59.92 ± 18.90 years, *p* = 0.020) and were more often female (48.4% vs. 39.0%, *p* = 0.037), White (86.3% vs. 69.8%, *p* < 0.001), and Hispanic/Latino (10.3% vs. 2.0%, *p* < 0.001). VAC patients more commonly presented with high-grade tumors (Grade 3: 64.7% vs. 32.6%, *p* < 0.001), advanced staged disease (Stage III/IV: 47.6% vs. 33.4%, *p* < 0.001), recurrent tumors (12.2% vs. 3.9%, *p* < 0.001), and with a history of unplanned excision (56.4% vs. 41.7%, *p* = 0.001). Tumor site varied (*p* = 0.007), with scalp tumors exclusively among VAC patients. Distribution of tumor size varied between groups, with the VAC group possessing proportionately less tumors smaller than 5 cm and less tumors greater than 10 cm (*p* = 0.033). Notably, tumor histology varied significantly between groups, with disproportionately more myxofibrosarcoma among VAC patients (48.4% vs. 25.4%, *p* < 0.001). VAC patients were more likely to receive radiation (67.6% vs. 34.2%, *p* < 0.001) and chemotherapy (15.4% vs. 3.6%, *p* < 0.001) and have shorter mean follow-up (38.99 ± 27.05 vs. 45.27 ± 25.35 months, *p* = 0.001). Reconstructive procedures were more common among VAC patients (flap reconstruction: 53.7% vs. 31.4%; skin graft: 31.9% vs. 20.5%, *p* = 0.001). Positive initial margins were more frequent in the VAC cohort (33.3% vs. 12.1%, *p* < 0.001), while negative final margins were more frequent among VAC patients, though not significantly (93.1% vs. 87.9%, *p* = 0.062). LR occurred in 16.0% of VAC patients compared to 4.8% of the comparison group (*p* < 0.001) (Table A1).

On univariate analysis, LR was associated with older age (69.09 ± 18.23 vs. 60.59 ± 17.67 years, *p* = 0.001), larger tumors (6.98 ± 5.17 vs. 5.40 ± 3.41 cm, *p* = 0.048), high-grade disease (Grade 3: 71.4% vs. 41.3%, *p* < 0.001), and a history of recurrent disease (19.6% vs. 5.5%, *p* = 0.002). Scalp (6.5% vs. 0.6%) and trunk tumors (15.2% vs. 10.2%) had high LR rates (*p* = 0.017), as did those requiring flap reconstruction (56.5% vs. 37.8%, *p* = 0.035) and those who received chemotherapy (19.6% vs. 6.8%, *p* = 0.006). Among LR patients, myxofibrosarcoma (43.5%) and undifferentiated pleomorphic sarcoma (UPS) (19.6%) were most frequent (*p* = 0.040). Additionally, advanced stage disease was more frequent among LR patients (Stage III/IV: 65.9% vs. 35.9%, *p* < 0.001). Tumor size was not associated with LR (*p* = 0.158). Positive initial margins were associated with LR (41.3% vs. 17.5%, *p* < 0.001), while final margin status (*p* = 0.12) and history of unplanned excision (*p* = 0.416) were not. Overall mortality (26.1% vs. 13.1%, *p* = 0.016) and metastasis (28.3% vs. 13.3%, *p* = 0.006) were higher among LR patients (Table A2).

After multivariate analysis, VAC use was associated with increased odds of LR (OR = 2.910; 95% CI, 1.408–6.014; *p* = 0.004). History of recurrent disease (OR = 3.080; 95% CI, 1.257–7.549; *p* = 0.014) and positive initial margin status (OR = 2.603; 95% CI, 1.287–5.265; *p* = 0.008) were also associated with LR odds. Radiation was protective, reducing odds of LR by about 50% (OR = 0.468; 95% CI 0.233–0.942; *p* = 0.033). Chemotherapy use (*p* = 0.136) and follow-up duration (*p* = 0.114) were not significantly associated (Table 1). Tumor grade was not included in multivariate analysis, because event frequencies were insufficient to support stable model estimation. No Grade 1 tumors experienced LR, and only twelve LR events occurred among Grade 2 tumors.

**Figure 1:**
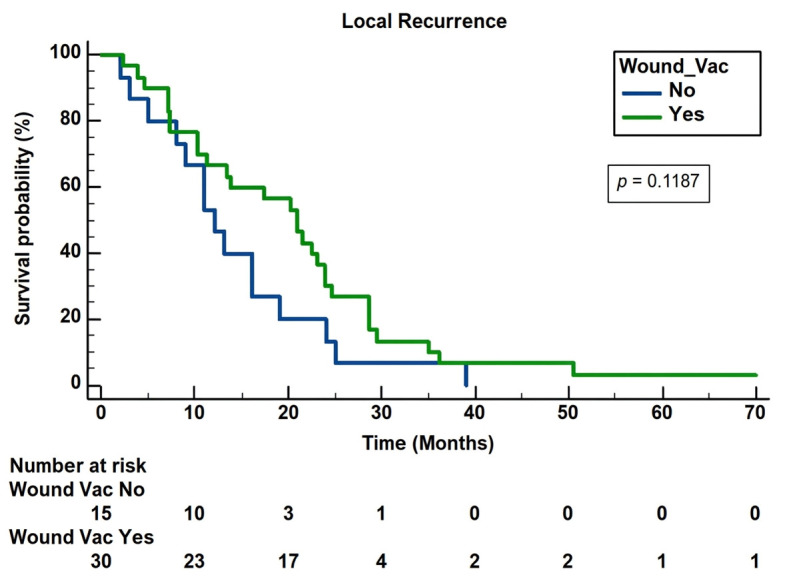
Kaplan–Meier analysis of LR-free survival, unmatched analysis.

Among patients who developed LR, median LR-free survival was 20.9 months (95% CI, 11.2–23.8) for VAC patients and 12 months (95% CI, 5.0–16.0) for non-VAC patients. However, this difference was not statistically significant (*p* = 0.12) (Figure 1).

### 3.2. Matched Cohort Analysis

After manual 1:1 matching by tumor grade, the sample size included 314 superficial STS, with 157 patients treated with VAC and 157 treated without VAC. Residual differences between cohorts persisted after matching. VAC patients were more often White (86.8% vs. 64.9%, *p* < 0.001) and Hispanic/Latino (10.5% vs. 1.4%, *p* = 0.001). Additionally, VAC patients were more likely to present with a history of prior unplanned excision (54.1% vs. 41.4%, *p* = 0.024) and with recurrent disease (12.7% vs. 3.8%, *p* = 0.004). Tumor histology remained significantly different between groups, with myxofibrosarcoma predominating among VAC-treated tumors, followed by undifferentiated pleomorphic sarcoma (UPS) (*p* < 0.001). Tumor size varied, with proportionately less tumors smaller than 5 cm and less tumors greater than 10 cm among the VAC cohort (*p* = 0.041). Age (*p* = 0.278), gender (*p* = 0.173), stage (*p* = 0.172), and site (*p* = 0.141) were similarly distributed after matching (Table 2).

VAC patients were more likely to receive radiation (73.2% vs. 38.5%, *p* < 0.001) and chemotherapy (14.6% vs. 6.4%, *p* = 0.017) and undergo flap reconstruction (56.7% vs. 35.0%, *p* < 0.001). Initial margins were more often positive among VAC patients (33.1% vs. 10.2%, *p* < 0.001), but final margin status did not differ significantly (*p* = 0.428). LR (16.6% vs. 5.1%, *p* = 0.001) was higher and overall mortality was lower (12.1% vs. 24.8%, *p* = 0.004) among VAC patients (Table 3).

On univariate analysis, LR was associated with older age (68.47 ± 19.92 vs. 62.64 ± 16.45 years, *p* = 0.013) higher-grade tumors (Grade 3: 82.4% vs. 60.0%, *p* = 0.029), and recurrent disease (23.5% vs. 6.4%, *p* = 0.003). Trunk tumors (26.1%) and scalp tumors (66.7%) demonstrated higher rates of LR (*p* = 0.024). Tumor size was not associated with LR (*p* = 0.268). Positive initial margins were associated with LR (19.1% vs. 8.5%, *p* = 0.013), while final margin status was not (*p* = 0.428). Tumor size (*p* = 0.102), flap reconstruction (*p* = 0.723), chemotherapy (*p* = 0.068), stage (*p* = 0.051), metastasis (*p* = 0.272), and overall mortality (*p* = 0.723) were no longer associated with LR after matching, though some trended toward significance. Among LR patients, myxofibrosarcoma (41.2%) and undifferentiated pleomorphic sarcoma (UPS) (23.5%) remained the most frequent diagnoses (*p* = 0.003). In total 34 LR events occurred, with 76.5% of LR patients deriving from the VAC cohort (*p* = 0.001) (Table 4).

In multivariate analysis of the matched cohort, temporizing VAC remained independently associated with increased LR (OR = 4.117; 95% CI 1.593–10.640; *p* = 0.003). Radiation therapy demonstrated about a 75% reduction in odds of LR (OR = 0.256; 95% CI, 0.109–0.602; *p* = 0.002). Patients presenting with recurrent disease had approximately 4 times higher odds of LR (OR = 4.003; 95% CI, 1.458–10.989; *p* = 0.007). Neither chemotherapy (*p* = 0.244) nor follow-up duration (*p* = 0.137) were associated with LR. Notably initial margin status was not independently associated with LR following multivariable adjustment (*p* = 0.114) (Table 5).

**Figure 2:**
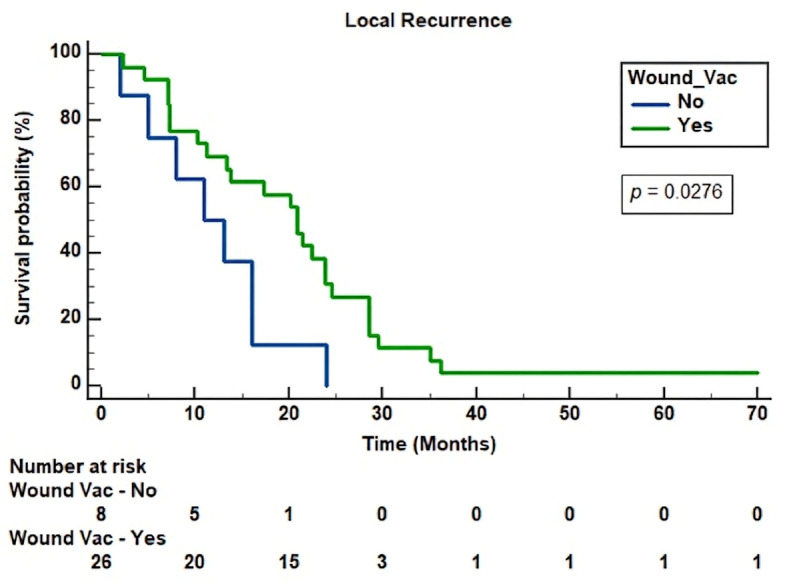
Kaplan–Meier analysis of LR-free survival, matched analysis.

Of the 34 LR patients, median LR-free survival was 20.9 months (95% CI, 11.2–23.8) for VAC patients and 11 months (95% CI, 2.0–16.0) for non-VAC patients. When time-to-event analyses were performed for the matched cohorts, the difference in median LR-free survival between the cohorts was statistically significant (*p* = 0.0276) (Figure 2).

## 4. Discussion

In this multi-institutional cohort, patients managed with staged resection and temporizing VAC represented a distinctly higher-risk clinical subgroup. Compared with patients treated without VAC, these individuals more frequently harbored high-grade tumors, advanced-staged disease, infiltrative histologies, and complex oncologic presentations. They were also more likely to have undergone prior unplanned excision or to present with recurrent disease. Furthermore, VAC patients tended to require more extensive soft-tissue reconstruction, reflecting both tumor- and treatment-related complexity. Collectively, these characteristics are well-established contributors to LR [20,21] and underscore the non-random clinical context in which staged resection with temporizing VAC is typically employed. Our analysis suggests that VAC use may be associated with increased time to LR; however, due to low event frequencies, we are likely underpowered to draw definitive conclusions.

Across both unmatched and grade-matched analyses, staged resection with temporizing VAC was not associated with improved LR rates. Although VAC use remained associated with higher LR rates after multivariable adjustment, this finding should not be interpreted as evidence that VAC therapy contributes causally to recurrence. Instead, VAC use appears to function as a marker of tumors with inherently aggressive biology, infiltrative growth patterns, and compromised oncologic margins: clinical scenarios in which the risk of LR may not be fully mitigated by surgical staging alone. With these limitations in mind, the intent of VAC therapy should be to facilitate margin assessment and staged reconstructive planning [22,23,24], not to alter tumor biology or compensate for biologically unfavorable disease.

Although tumor diameter did not differ between cohorts, the significantly higher rate of flap reconstruction among the VAC cohort likely reflects the combined influence of infiltrative histology (predominantly myxofibrosarcoma), higher rates of prior unplanned excision, and recurrent disease necessitating wider re-excision, greater use of preoperative radiation compromising primary closure, and institutional differences in reconstructive practice. This suggests that tumor diameter alone is an incomplete measure of surgical defect complexity, as tumor depth, volume, growth pattern, and the extent of the required re-excision field all contribute to reconstructive decision-making.

Notably, initial positive margin status was associated with LR in the unmatched analysis but not after grade-based matching, likely reflecting reduced statistical power and collinearity with tumor grade, histology, and presentation status. These further findings highlight the complex interplay between surgical margins and underlying tumor biology [25,26]. In certain cases, positive margins may reflect diffuse microscopic contamination or infiltrative tumor behavior rather than a technically correctable surgical error [27,28]. Although a central purpose of staged resection is to facilitate final negative margins, particularly for high-risk clinical scenarios such as myxofibrosarcoma or prior unplanned excision, we found that final margin status did not differ between cohorts and was not associated with LR in either analysis. This suggests VAC-facilitated re-excision may be insufficient to meaningfully alter recurrence risk, despite achieving negative margins.

Consistent with prior literature, radiation demonstrated a protective association against LR across multiple analyses [29,30]. Temporizing VAC may be able to facilitate adjuvant radiotherapy by better demarcating the tumor bed and reducing overall toxicity [31], particularly in highly infiltrative STS. Additionally, higher rates of metastasis and mortality were observed among patients who developed LR in the unmatched cohort, aligning with established associations linking local failure to systemic progression [5,6]. The attenuation of these associations after matching further suggests that adverse oncologic outcomes are driven predominantly by underlying tumor biology rather than LR alone [28].

Taken together, these findings reinforce that temporizing VAC should be viewed primarily as a reconstructive and logistical strategy rather than an oncologic intervention. While temporizing VAC may offer advantages in managing complex wounds and facilitating staged reconstruction, its use should not substitute for meticulous preoperative planning, careful assessment of tumor extent, and adherence to sound oncologic surgical principles.

### Limitations

This study is limited by its retrospective design and relatively low event rates despite multi-institutional collaboration, which reduced statistical power and constrained multivariable analysis. Despite restricting the cohorts to superficial tumors, differences in tumor biology, presentation status, and institutional practices persisted between treatment groups, reflecting the non-random selection of patients for VAC therapy and introducing confounding by indication. Follow-up duration, while comparable to prior literature, may be insufficient to capture late recurrences and establish comparison of long-term recurrence rates. Limited recurrence events and small subgroups also precluded robust adjustment for tumor grade and histologic subtype (Tables S1 and S2). Notably, myxofibrosarcoma, which carries an inherently higher risk of LR, was disproportionately represented in the VAC cohort and may have confounded the observed association. Surgeon-specific decision-making, margin assessment techniques, and institutional differences in pathology turnaround time were not captured and may have influenced both the use of VAC and oncologic outcomes. Finally, the absence of standardized criteria for selecting patients for staged resection with VAC introduces variability that may limit generalizability. Prospective studies or consensus-driven guidelines are needed to better define which patients may derive the greatest benefit from this approach.

Despite these limitations, this multi-institutional study provides one of the broadest evaluations of temporizing VAC across the spectrum of superficial STS. Our findings suggest that VAC use may be most valuable in facilitating complex soft tissue reconstruction and radiation therapy rather than mitigating oncologic outcomes.

## 5. Conclusions

• Staged resection with temporizing VAC was not associated with improved LR rates, with the VAC cohort possessing intrinsically higher-risk tumor biology and complex oncologic presentations.
• LR risk in such complex patients may not be fully mitigated by surgical staging.
• VAC use should be viewed as an adjunct for complex reconstructive planning and facilitation of radiation therapy.
• Future efforts should focus on establishing standardized indications for staged resection in STS surgery.

## Figures and Tables

**Table 1:** Association of LR after Univariate and Multivariate Analysis for unmatched analysis.

	Univariate Analysis	Multivariate Analysis	
Variable	OR (95% CI)	OR (95% CI)	*p*-Value
Temporizing VAC	3.738 (1.979, 7.062)	2.910 (1.408, 6.014)	0.004
Presenting Status	4.018 (1.760, 9.174)	3.080 (1.257, 7.549)	0.014
Chemotherapy	3.352 (1.488, 7.551)	1.954 (0.810, 4.717)	0.136
Radiation	0.881 (0.479, 1.622)	0.468 (0.233, 0.942)	0.033
Follow Up	0.981 (0.967, 0.995)	0.988 (0.974, 1.003)	0.114
Initial Margin	3.307 (1.756, 6.227)	2.603 (1.287, 5.265)	0.008

**Table 2:** Demographics and tumor information of matched analysis.

		Non-VAC (*n* = 157)	VAC (*n* = 157)	
Variable		*n* (%) or Mean ± SD	*n* (%) or Mean ± SD	*p*-Value
Age (years)		62.4 ± 18.35	64.50 ± 15.31	0.278
Gender	Male	93 (59.2)	81 (51.6)	0.173
	Female	64 (40.8)	76 (48.4)	
Race	White	96 (64.9)	131 (86.8)	<0.001
	Non-White	52 (35.1)	20 (13.2)	
Ethnicity	Hispanic/Latino	2 (1.4)	16 (10.5)	0.001
	Non-Hispanic/Latino	137 (98.6)	137 (89.5)	
Tumor diameter (cm)		6.02 ± 4.04	6.03 ± 3.52	0.551
Tumor diameter	Less than 5 cm	80 (54.4)	71 (46.1)	0.041
	5–10 cm	45 (30.6)	68 (44.2)	
	Greater than 10 cm	22 (15.0)	15 (9.7)	
Grade	1	9 (5.7)	9 (5.7)	1
	2	50 (31.8)	50 (31.8)	
	3	98 (62.4)	98 (62.4)	
Stage	1	8 (5.4)	14 (9)	0.172
	2	76 (51.7)	67 (42.9)	
	3	61 (41.5)	68 (43.6)	
	4	2 (1.4)	7 (4.5)	
Presenting status	Primary	151 (96.2)	137 (87.3)	0.004
	Recurrent	6 (3.8)	20 (12.7)	
Site	Upper extremity	50 (31.8)	38 (24.2)	0.141
	Lower extremity	91 (58)	102 (65)	
	Trunk	16 (10.2)	13 (8.3)	
	Scalp	0 (0)	3 (1.9)	
Prior unplanned excision	No	92 (58.6)	72 (45.9)	0.024
	Yes	65 (41.4)	85 (54.1)	
Diagnosis	Myxofibrosarcoma	43 (27.4)	85 (54.1)	<0.001
	UPS	23 (14.6)	19 (12.1)	
	Leiomyosarcoma	28 (17.8)	9 (5.7)	
	Fibrosarcoma	7 (4.5)	1 (0.6)	
	Other STS	56 (35.7)	43 (27.4)	

**Table 3:** Treatment information and outcomes of matched analysis.

		Non-VAC (*n* = 157)	VAC (*n* = 157)	
Variable		*n* (%) or Mean ± SD	*n* (%) or Mean ± SD	*p*-Value
Initial margin status	Negative	141 (89.8)	105 (66.9)	<0.001
	Positive	16 (10.2)	52 (33.1)	
Final margin status	Negative	141 (89.8)	145 (92.4)	0.428
	Positive	16 (10.2)	12 (7.6)	
Radiation	No	96 (61.5)	42 (26.8)	<0.001
	Yes	60 (38.5)	115 (73.2)	
Chemotherapy	No	147 (93.6)	134 (85.4)	0.017
	Yes	10 (6.4)	23 (14.6)	
Soft tissue coverage	Primary closure	65 (41.4)	20 (12.7)	<0.001
	Skin graft	37 (23.6)	48 (30.6)	
	Flap reconstruction	55 (35)	89 (56.7)	
Follow up		42.94 ± 25.66	40.60 ± 27.99	0.165
LR	No	149 (94.9)	131 (83.4)	0.001
	Yes	8 (5.1)	26 (16.6)	
Metastasis	No	123 (78.3)	130 (82.8)	0.318
	Yes	34 (21.7)	27 (17.2)	
Time to metastasis (months)		20.06 ± 26.55	17.00 ± 17.70	0.89
Overall survival	Alive	118 (75.2)	138 (87.9)	0.004
	Dead	39 (24.8)	19 (12.1)	

**Table 4:** Distribution and associations of LR for matched analysis.

		No LR (*n* = 280)	LR (*n* = 34)	
Variable		*n* (%) or Mean ± SD	*n* (%) or Mean ± SD	*p*-Value
Age (years)		62.64 ± 16.45	68.47 ± 19.92	0.013
Gender	Male	151 (53.9)	23 (67.6)	0.129
	Female	129 (46.1)	11 (32.4)	
Race	White	199 (74.8)	28 (84.8)	0.203
	Non-White	67 (25.2)	5 (15.2)	
Ethnicity	Hispanic/Latino	14 (5.4)	4 (12.5)	0.12
	Non-Hispanic/Latino	246 (94.6)	28 (84.8)	
Tumor diameter (cm)		5.84 ± 3.44	7.51 ± 5.71	0.189
Tumor diameter	Less than 5 cm	137 (51.1)	14 (42.4)	0.268
	5–10 cm	101 (37.7)	12 (36.4)	
	Greater than 10 cm	30 (11.2)	7 (21.2)	
Grade	1	18 (6.4)	0 (0)	0.029
	2	94 (33.6)	6 (17.6)	
	3	168 (60)	28 (82.4)	
Stage	1	21 (7.8)	1 (3)	0.051
	2	133 (49.3)	10 (30.3)	
	3	109 (40.4)	20 (60.6)	
	4	7 (2.6)	2 (6.1)	
Presenting status	Primary	262 (93.6)	26 (76.5)	0.003
	Recurrent	18 (6.4)	8 (23.5)	
Site	Upper extremity	81 (28.9)	7 (20.6)	0.024
	Lower extremity	174 (62.1)	19 (55.9)	
	Trunk	23 (8.2)	6 (17.6)	
	Scalp	1 (0.4)	2 (5.9)	
Prior unplanned excision	No	143 (51.1)	21 (61.8)	0.238
	Yes	137 (48.9)	13 (38.2)	
Diagnosis	Myxofibrosarcoma	114 (40.7)	14 (41.2)	0.003
	UPS	34 (12.1)	8 (23.5)	
	Leiomyosarcoma	35 (12.5)	2 (5.9)	
	Fibrosarcoma	7 (2.5)	1 (2.9)	
	Other STS	90 (32.1)	9 (26.5)	
Initial margin status	Negative	225 (80.4)	21 (61.8)	0.013
	Positive	55 (19.6)	13 (38.2)	
Final margin status	Negative	257 (91.8)	29 (85.3)	0.206
	Positive	24 (8.2)	5 (14.7)	
Radiation	No	118 (42.3)	20 (58.8)	0.067
	Yes	161 (57.7)	14 (41.2)	
Chemotherapy	No	254 (90.7)	27 (79.4)	0.068
	Yes	26 (9.3)	7 (20.6)	
Soft tissue coverage	Primary closure	78 (27.9)	7 (20.6)	0.275
	Skin graft	78 (27.9)	7 (20.6)	
	Flap reconstruction	124 (44.3)	20 (58.8)	
Follow up		43.02 ± 26.95	31.48 ± 23.77	0.009
VAC	No	149 (53.2)	8 (23.5)	0.001
	Yes	131 (46.8)	26 (76.5)	
Metastasis	No	228 (81.4)	25 (73.5)	0.272
	Yes	52 (18.6)	9 (26.5)	
Time to metastasis (months)		17.89 ± 22.83	24.31 ± 25.54	0.542
Overall survival	Alive	231 (82.5)	25 (73.5)	0.203
	Dead	49 (17.5)	9 (26.5)	

**Table 5:** Association of LR after Univariate and Multivariate Analysis for matched analysis.

	Univariate Analysis	Multivariate Analysis	
Variable	OR (95% CI)	OR (95% CI)	*p*-Value
Temporizing VAC	3.697 (1.618, 8.447)	4.117 (1.593, 10.640)	0.003
Radiation	0.513 (0.249, 1.057)	0.256 (0.109, 0.602)	0.002
Initial Margin	2.532 (1.194, 5.371)	1.995 (0.848, 4.695)	0.114
Chemotherapy	2.533 (1.005, 6.381)	1.819 (0.665, 4.972)	0.244
Follow Up	0.980 (0.964, 0.997)	0.986 (0.969, 1.004)	0.137
Presenting Status	4.479 (1.776, 11.297)	4.003 (1.458, 10.989)	0.007

## Data Availability

The data is stored internally at Moffitt Cancer Center, Massachusetts General Hospital, and Mount Sinai Hospital. If there is interest in obtaining the data, please contact the study team.

## Supplementary Materials

The following supporting information can be downloaded at: https://www.mdpi.com/article/10.3390/cancers18121984/s1, Table S1: Full distribution of tumor histology, LR, and VAC treatment for unmatched analysis; Table S2: Full distribution of tumor histology, LR, and VAC treatment for matched analysis.

## Abbreviations

The following abbreviations are used in this manuscript:

VAC	Vacuum-assisted closure
STS	Soft tissue sarcoma
LR	Local recurrence
DFSP	Dermatofibrosarcoma protuberans
UPS	Undifferentiated pleomorphic sarcoma

## Appendix A

**Table A1:** Demographics, tumor and treatment information, and outcomes of unmatched analysis.

		Non-VAC (*n* = 331)	VAC (*n* = 188)	
Variable		*n* (%) or Mean ± SD	*n* (%) or Mean ± SD	*p*-Value
Age (years)		59.92 ± 18.90	63.86 ± 15.62	0.02
Gender	Male	202 (61)	97 (51.6)	0.037
	Female	129 (39)	91 (48.4)	
Race	White	215 (69.8)	157 (86.3)	<0.001
	Non-White	93 (30.2)	25 (13.7)	
Ethnicity	Hispanic/Latino	6 (2)	19 (10.3)	<0.001
	Non-Hispanic/Latino	298 (98)	165 (89.7)	
Tumor diameter (cm)		5.46 ± 3.72	5.69 ± 3.48	0.226
Tumor diameter	Less than 5 cm	178 (65.7)	93 (50.5)	0.033
	5–10 cm	90 (29.5)	75 (40.8)	
	Greater than 10 cm	37 (12.1)	16 (8.7)	
Grade	1	71 (23.6)	9 (5.4)	<0.001
	2	132 (43.9)	50 (29.9)	
	3	98 (32.6)	108 (64.7)	
Stage	1	65 (23.3)	14 (8.4)	<0.001
	2	121 (43.4)	73 (44)	
	3	90 (32.3)	72 (43.4)	
	4	3 (1.1)	7 (4.2)	
Presenting status	Primary	318 (96.1)	165 (87.8)	<0.001
	Recurrent	13 (3.9)	23 (12.2)	
Site	Upper extremity	106 (32)	49 (26.2)	0.007
	Lower extremity	189 (57.1)	114 (60.4)	
	Trunk	36 (10.9)	19 (10.2)	
	Scalp	0 (0)	6 (3.2)	
Prior unplanned excision	No	193 (58.3)	82 (43.6)	0.001
	Yes	138 (41.7)	106 (56.4)	
Diagnosis	Myxofibrosarcoma	84 (25.4)	91 (48.4)	<0.001
	UPS	34 (10.3)	22 (11.7)	
	Leiomyosarcoma	68 (20.5)	9 (4.8)	
	Fibrosarcoma	21 (6.3)	1 (0.5)	
	Other STS	124 (37.5)	65 (34.6)	
Initial margin status	Negative	291 (87.9)	126 (67)	<0.001
	Positive	40 (12.1)	62 (33)	
Final margin status	Negative	291 (87.9)	175 (93.1)	0.062
	Positive	40 (12.1)	13 (6.9)	
Radiation	No	218 (65.8)	61 (32.4)	<0.001
	Yes	113 (34.2)	127 (67.6)	
Chemotherapy	No	319 (96.4)	159 (84.6)	<0.001
	Yes	12 (3.6)	29 (15.4)	
Soft tissue coverage	Primary closure	159 (48)	27 (14.4)	0.001
	Skin graft	68 (20.5)	60 (31.9)	
	Flap reconstruction	104 (31.4)	101 (53.7)	
Follow up		45.27 ± 25.35	38.99 ± 27.05	0.001
LR	No	315 (95.2)	158 (84)	<0.001
	Yes	16 (4.8)	30 (16)	
Metastasis	No	284 (85.8)	159 (84.6)	0.704
	Yes	47 (14.2)	29 (15.4)	
Time to metastasis (months)		22.77 ± 27.58	17.07 ± 17.03	0.674
Overall survival	Alive	278 (84)	167 (88.8)	0.129
	Dead	53 (16)	21 (11.2)	

**Table A2:** Distribution and association of LR for unmatched analysis.

		No LR (*n* = 473)	LR (*n* = 46)	
Variable		*n* (%) or Mean ± SD	*n* (%) or Mean ± SD	*p*-Value
Age (years)		60.59 ± 17.67	69.09 ± 18.23	0.001
Gender	Male	267 (56.4)	32 (69.6)	0.086
	Female	206 (43.6)	14 (30.4)	
Race	White	337 (75.6)	35 (79.5)	0.555
	Non-White	109 (24.4)	9 (20.5)	
Ethnicity	Hispanic/Latino	21 (4.7)	4 (9.3)	0.263
	Non-Hispanic/Latino	424 (95.3)	39 (90.7)	
Tumor diameter (cm)		5.40 ± 3.41	6.98 ± 5.17	0.048
Tumor diameter	Less than 5 cm	251 (56.5)	20 (44.4)	0.158
	5–10 cm	148 (33.3)	17 (37.8)	
	Greater than 10 cm	45 (10.1)	8 (17.8)	
Grade	1	80 (18.8)	0 (0)	<0.001
	2	170 (39.9)	12 (28.6)	
	3	176 (41.3)	30 (71.4)	
Stage	1	78 (19.3)	1 (2.4)	<0.001
	2	181 (44.8)	13 (31.7)	
	3	137 (33.9)	25 (61)	
	4	8 (2)	2 (4.9)	
Presenting status	Primary	446 (94.3)	37 (80.4)	0.002
	Recurrent	26 (5.7)	9 (19.6)	
Site	Upper extremity	144 (30.5)	11 (23.9)	0.017
	Lower extremity	277 (58.7)	25 (54.3)	
	Trunk	48 (10.2)	7 (15.2)	
	Scalp	3 (0.6)	3 (6.5)	
Prior unplanned excision	No	248 (52.4)	27 (58.7)	0.416
	Yes	225 (47.6)	19 (41.3)	
Diagnosis	Myxofibrosarcoma	155 (32.8)	20 (43.5)	0.040
	UPS	47 (9.9)	9 (19.6)	
	Leiomyosarcoma	75 (15.9)	2 (4.3)	
	Fibrosarcoma	21 (4.4)	1 (2.2)	
	Other STS	175 (37)	14 (30.4)	
Initial margin status	Negative	390 (82.5)	27 (58.7)	<0.001
	Positive	83 (17.5)	19 (41.3)	
Final margin status	Negative	428 (90.5)	38 (82.6)	0.12
	Positive	45 (9.5)	8 (17.4)	
Radiation	No	252 (53.4)	26 (56.5)	0.684
	Yes	220 (46.6)	20 (43.5)	
Chemotherapy	No	441 (93.2)	37 (80.4)	0.006
	Yes	32 (6.8)	9 (19.6)	
Soft tissue coverage	Primary closure	176 (37.2)	10 (21.7)	0.035
	Skin graft	118 (24.9)	10 (21.7)	
	Flap reconstruction	179 (37.8)	26 (56.5)	
Follow up		43.96 ± 26.18	33.04 ± 23.64	0.003
VAC	No	315 (66.6)	16 (34.8)	<0.001
	Yes	158 (33.4)	30 (65.2)	
Metastasis	No	410 (86.7)	33 (71.7)	0.006
	Yes	63 (13.3)	13 (28.3)	
Time to metastasis (months)		20.24 ± 24.92	23.03 ± 21.56	0.359
Overall survival	Alive	411 (86.9)	34 (73.9)	0.016
	Dead	62 (13.1)	12 (26.1)	
