# Supplementary material for: Staged Resection with Temporizing VAC and Local Recurrence for Soft Tissue Sarcomas: A Multi-Institutional Review
Source: Cancers (Basel). 2026 Jun 18;18(12):1984. doi: 10.3390/cancers18121984 (PMC13297323; doi:10.3390/cancers18121984)
Supplement: Supplementary file 1 [file cancers-18-01984-s001.zip › cancers-4375529-supplementary.pdf]

Supplemental Material

Additional Tables

Table S1. Full distribution of tumor histology, LR, and VAC treatment for unmatched analysis.

		No VAC (n=331)	VAC (n=188)
**Diagnosis**	**Local Recurrence**	**N (%)**	**N (%)**
Myxofibrosarcoma	No	77 (91.7)	78 (85.7)
	Yes	7 (8.3)	13 (14.3)
Undifferentiated pleomorphic sarcoma	No	31 (91.2)	16 (72.7)
	Yes	3 (8.8)	6 (27.3)
Leiomyosarcoma	No	68 (100)	7 (77.8)
	Yes	0 (0)	2 (22.2)
Fibrosarcoma	No	20 (95.2)	1 (100)
	Yes	1 (4.8)	0 (0)
Dermatofibrosarcoma protuberans	No	1 (100)	13 (92.9)
	Yes	0 (0)	1 (7.1)
Myxoid liposarcoma	No	12 (100)	4 (100)
Pleomorphic liposarcoma	No	5 (100)	1 (100)
Dedifferentiated liposarcoma	No	9 (100)	3 (60)
	Yes	0 (0)	2 (40)
Myxoid and round cell liposarcoma	No	1 (100)	0 (0)
Myofibrosarcoma	No	1 (100)	0 (0)
Fibromyxoid sarcoma	No	7 (100)	1 (100)
Synovial sarcoma	No	2 (66.7)	5 (100)
	Yes	1 (33.3)	0 (0)
Angiosarcoma	No	1 (100)	2 (50)
	Yes	0 (0)	2 (50)
Epithelioid sarcoma	No	4 (100)	2 (66.7)
	Yes	0 (0)	1 (33.3)
Hemangiopericytoma – solitary fibrous tumor	No	7 (87.5)	0 (0)
	Yes	1 (12.5)	0 (0)
Rhabdomyosarcoma	No	4 (100)	3 (50)
	Yes	0 (0)	3 (50)
Malignant peripheral nerve sheath tumor	No	10 (100)	3 (100)
Soft tissue osteosarcoma	No	4 (100)	5 (100)
Soft tissue chondrosarcoma	No	1 (100)	1 (100)
Ewing’s sarcoma	No	6 (85.7)	1 (100)
	Yes	1 (14.3)	0 (0)
Clear cell sarcoma	No	2 (100)	0 (0)
STS – not otherwise specified	No	42 (95.5)	12 (100)
	Yes	2 (4.5)	0 (0)

Table S2. Full distribution of tumor histology, LR, and VAC treatment for matched analysis.

		No VAC (n=157)	VAC (n=157)
**Diagnosis**	**Local Recurrence**	**N (%)**	**N (%)**
Myxofibrosarcoma	No	41 (95.3)	73 (85.9)
	Yes	2 (4.7)	12 (14.1)
Undifferentiated pleomorphic sarcoma	No	21 (91.3)	13 (68.4)
	Yes	2 (8.7)	6 (31.6)
Leiomyosarcoma	No	28 (100)	7 (77.8)
	Yes	0 (0)	2 (22.2)
Fibrosarcoma	No	6 (85.7)	1 (100)
	Yes	1 (14.3)	0 (0)
Dermatofibrosarcoma protuberans	No	1 (100)	2 (100)
Myxoid liposarcoma	No	2 (100)	3 (100)
Pleomorphic liposarcoma	No	2 (100)	1 (100)
Dedifferentiated liposarcoma	No	4 (100)	3 (60)
	Yes	0 (0)	2 (40)
Myxoid and round cell liposarcoma	No	1 (100)	0 (0)
Myofibrosarcoma	No	1 (100)	0 (0)
Synovial sarcoma	No	0 (0)	5 (100)
Angiosarcoma	No	0 (0)	1 (50)
	Yes	0 (0)	1 (50)
Epithelioid sarcoma	No	0 (0)	1 (50)
	Yes	0 (0)	1 (50)
Hemangiopericytoma – solitary fibrous tumor	No	3 (100)	0 (0)
Rhabdomyosarcoma	No	4 (100)	3 (60)
	Yes	0 (0)	2 (40)
Malignant peripheral nerve sheath tumor	No	5 (100)	3 (100)
Soft tissue osteosarcoma	No	3 (100)	5 (100)
Soft tissue chondrosarcoma	No	0 (0)	1 (100)
Ewing's sarcoma	No	6 (85.7)	0 (0)
	Yes	1 (14.3)	0 (0)
Clear cell sarcoma	No	2 (100)	0 (0)
STS – not otherwise specified	No	18 (90)	8 (100)
	Yes	2 (10)	0 (0)
